# Phenomenal Case of Hysteria

**Published:** 1887-07

**Authors:** F. A. Schmitt

**Affiliations:** Schulenburg, Texas


### PHENOMENAL CASE OF HYSTERIA.

*By F. A. Schmitt, M. D. Schulenburg, Texas.*

For Daniel's Texas Medical Journal.

THE case of hysteria reported in the March number of your JOURNAL, by Dr. W. M. Powell, of Albany, Texas, illustrates vividly the condition of a young lady during an acute attack of malady lasting two days. The similarity of reported case and the one which is made the subject of this paper is remarkable, differing only as far as regards duration and multiplicity of symptoms.

As the name implies, is hysteria supposed to have its seat in the uterus? Certain it is that lesions of the sexual organs produce similar symptoms, and that with the re-established normal condition of these organs these symptoms disappear.

Undoubtedly are there cases on record in which the womb has taken no share in producing it.

I remember one plain case of hysteria in a male. Hereditary predisposition, combined with defective training, may also cause this anomaly, as the case taken from my records, and below narrated, will doubtless prove; and how varied and manifold the symptoms of what the term implies may become, was again impressed on my mind in looking over this record.

Disturbances of sensibility, of mobility, of the psychic functions and disturbances in the compass of the vaso-motor and trophical nerves (Niemeier—Pathol. and Theraptcs.) may each, for itself, or in conjunction, be present in such poor sufferers, who, among the laiety, may even be denied that sympathy other victims of disease generally receive—as in the case under consideration—no doubt owing to the seemingly feigned, and to all appearances, trifling abnormalities present, or in consequence of the rarely or never occurring fatal termination of such cases.

Childless wives, widows and old maids, seem to be more subject to this anomaly then others, but that occasionally bad, and the worst cases occur among females at beginning or instituted puberty has been my experience, and made apparent in the case above alluded to; a case as peculiar in its varied symptoms and violent outbursts, as it is deplorable and interesting to the practitioner of medicine. A real psychological enigma, mysterious in the extreme.

A female in the bloom of youth is made an invalid, is stricken down and confined to bed for years on account of this ailment and without demonstrable cause,—certainly without positive signs of organic lesion of the uterus or other organ.

With this patient—but under certain local conditions only—the whole train of hysterical symptoms follow each other in rapid succession, culminating at times in a hyperæsthesia of the general integument, or pains confined to certain parts of the body the (apparent) severity of which it is hardly possible to describe.

A more wretched, pitiable condition I have not often been called upon to witness, and to alleviate, as her condition during paroxysms of pain in the region of the sagital suture (clavus hystericus) or while affecting a joint (anthropathia hysterica).

As I remarked before; is this condition in her induced, aggravated or prolonged by a certain stimulus, and remarkable in the extreme is the fact, that this irritant—I do not know how else to term it—consisted and consists up to this day,—in the presence, as nurse, *of her mother*, or more correctly perhaps, in the general surroundings or peculiarities of her paternal domicile. This irritant or stimulus induces in the girl a complete loss of selfcontrol,—a veritable psychosis.—and this in turn causes the pathological condition below more in detail described.

For years, as already stated, has this otherwise bright and intelligent girl—with but short intervals, and then among strangers—been confined to her bed, suffering all the while as indicated. Only after forcibly being placed among strangers—her medical attendants—will she, after a lapse of days or weeks, and then requiring all the energy, skill and cunning at the command of the attendants, regain sufficient self control to move and behave as becoming one of her sex, with sound body and mind; and every attempt of returning her to her parental roof has inevitably been followed by a relapse.

She had been in charge of a professional friend, who, on several occasions, had discharged her cured. During one of these relapses, two years ago, I was consulted, but as the case had been so successfully treated by her former attendant, I advised the parents to again put her under his care; and only after pressing the matter, and expressing their wish of having her cured at home, and then perhaps for good, did I consent to try the experiment.

My first visit made me a witness of her condition during one of her worst attacks; the mother and daughter being perfectly exhausted, the former from her endeavor by friction of back, arms and legs of the girl to give her temporary ease, and the latter, in consequence of pain (imagined or not), of restlessness and cramps.

My arrival changed the scene somewhat. The girl had been informed of a new doctor to take charge of her, and my presence seemed to have a quieting effect. She submitted quietly to an examination of her body, showed her tongue, admitted the thermometer in the axilla, allowed me to place the stethoscope over the regions of heart and lungs and answered questions readily and cheerfully, all of which—as I was informed—she generally persistently refused during her spells. I found her pulse slightly increased in number (85 per minute), the hearts action steady and uniform, her temperature little above the normal (98½), bowels regular, appetite good, had had her monthly one week previous, and periodically before that, at regular intervals since puberty; does not change and has not before, materially changed her condition. Her extremities were cold, and her body covered with a profuse, clammy perspiration; had severe pain in left ankle (no swelling) and was unable to rise from a horizontal position and had been since her arrival home three weeks before.

Failing to detect organic lesions, and my questions in regard to morbid habits (!) being satisfactorily answered by her constant attendant, I attributed her condition to chlorosis—color of skin and lips corroborating. For immediate relief I ordered aromatic fomentations to ankle, and a tea spoon full every 2 hours of a mixture of Extr. Valer. fl. and chloral hydrate.

Called again several hours afterwards and found her comparatively well and free of pain; had slept during my absence. Prescribed Iron in the form of Blaud's pills: ℞. Ferr. Sulph. Potass. Carb. aa. ℥ ss—Gum. Tragacanth, g s. ft. pill No. IVC. 3 to 5 pills 3 times during the 24 hours, and recommended general tonic treatment.

From then on I called regularly, (once daily) four consecutive weeks.

During first week she improved visibly; she had had but one attack since my first visit, the second day, began to sit up in bed; became cheerful, wished to read, and I entertained hopes, and her also, of her ultimate perfect restoration.

During the second week, although the iron taken in large quantities (and which she seemed to crave), began to tell on her complexion, she again relapsed. Hyperæsthesia and, at times, total anæsthesia, of parts of her body, became complete. Pain here and there and everywhere, but more especially acute in the neighborhood of the sagital suture or near joints, convulsions, choking sensations (bolus hystericus), and all the varied symptoms constituting this obstinate malady again became the order of the day; mother and patient now almost despairing. Constantly was her parent engaged in rubbing and brushing body and extremities of the patient to ease her, if only for a short period, exhausting herself as well as her child.

The requests of the girl for these "rubbings" I had, on several occasions, overheard on entering the premises; they were given either in a pitiful, begging tone, or cammandingly, not unlike the commands of a general in the field; the result being invariably the same. Immediately would the mother, armed with brush or towel, repair to her post and proceed with her rubbing, brushing or kneading much like the massage craze of to-day—until complete prostration put a stop to the parents exertions. I explained the folly and the possibie harm of this tiresome practice. I implored, I begged her to abstain from it. I commanded her to desist and to but strictly conform to directions given, and no more—all to no purpose. Was it sympathy with her offspring, was it craze or hysterical "want of reason" with her also which induced her to admit of being imposed on continually—I am not prepared to say. Certain it is that the soothing effect of my daily visit upon the patient was more than counterbalanced by the ill-timed and unreasonable regard the mother displayed concerning this morbid desire of her daughter.

Finding my intentions thus thwarted, and no other remedy except separation of patient and nurse, I proposed to take her to my own home for treatment, but on account of sickness in my family this was delayed.

Shortly afterwards, stretcher, ambulance and other parapharnalia for transporting the sick were necessary requisites for her "displacement," and she was returned to her former medical attendant, where she—but only after a hard "psychical" struggle—again regained her self-control, and where she, up to the present time, enjoyed comparative health.

Evidently is this unfortunate girl a victim of hereditary predisposition to nervous hyper-sensibility; and if we add to this presumably defective training and unwholesome literature, we may then come as near reckoning the causes for her troubles as it it possible for us to do.

